# A giant calcified aneurysm of the basal inferior wall: a rare phenomenon

**DOI:** 10.11604/pamj.2020.37.193.26214

**Published:** 2020-10-29

**Authors:** Emna Bennour, Ahmed Sghaier, Amine Jemel, Lobna Laroussi, Kamoun Ikram, Sonia Marrakchi, Neji Henda, Kachboura Salem

**Affiliations:** 1 Cardiology Department, Abderrahmen Mami University Hospital, Ariana, Tunis, Tunisia,; 2 Cardiothoracic Surgery Department, Abderrahmen Mami University Hospital, Ariana, Tunis, Tunisia,; 3 Radiology Department, Abderrahmen Mami University Hospital, Ariana, Tunis, Tunisia

**Keywords:** Aneurysm, left ventricle, basal wall, myocardial infarction

## Abstract

Left ventricular aneurysms (LVA) are mainly a late consequence of transmural myocardial infarction. Approximately 80% of LVA are located in the anterior and/or apical walls, most commonly associated with left anterior descending artery occlusion but any region may be engaged. Basal inferior wall aneurysms are rare and constitute nearly 3% of all LVA. A calcified LVA is seldom observed in modern clinical practice. And a calcified basal inferior LVA is an even rarer coincidence. We report a case of an 82-year-old women with life threatening arrhythmia revealing a giant calcified aneurysm of the basal inferior wall, medically treated with good outcomes. The exact incidence of left ventricular aneurysms (LVA) following myocardial infarctions is hard to precise but it is clearly decreasing. Eighty percent (80%) of LVA are located in the anterior or apical walls, but any region may be engaged. Basal inferior wall aneurysms constitute 3% of all LVA. Echocardiography is the first diagnostic tool and there is still no clear guidelines on how to treat LVAs. Surgery is preferred but medical treatment may help improve the quality of life.

## Introduction

Left ventricular aneurysms (LVA) are mainly a late consequence of transmural myocardial infarction (MI) but besides atherosclerotic coronary artery disease, other uncommon causes of LVA include myocarditis, hypertrophic cardiomyopathy, chest trauma, arrhythmogenic right ventricular dysplasia, Chaga´s disease, HIV, and connective tissue diseases like sarcoidosis and systemic lupus. Idiopathic LVA exists but are very rare [1]. Although the exact definition of LVA remains controversial, it is usually defined as a thin, akinetic or dyskinetic and scarred or fibrotic wall, devoid of muscle or containing necrotic muscle as a consequence of transmural MI [2].

## Patient and observation

**Figure 1:**
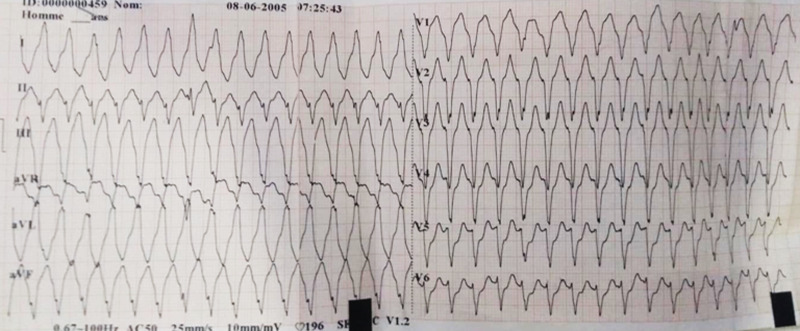
ECG showing a wide complex tachycardia at 200bpm with a superior left axis

**Figure 2:**
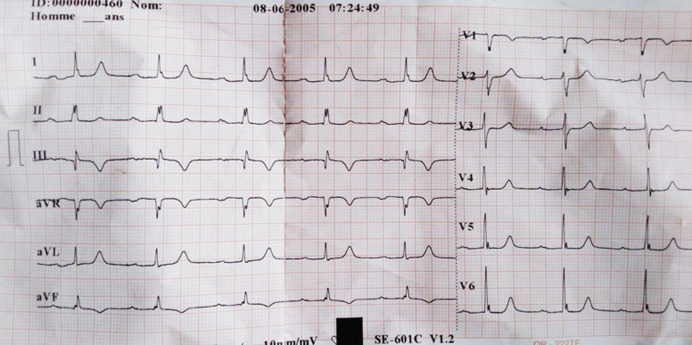
ECG after electrical cardioversion showing a first degree AV block with Q waves in DIII, inverted T waves in leads AVF and D3

**Figure 3:**
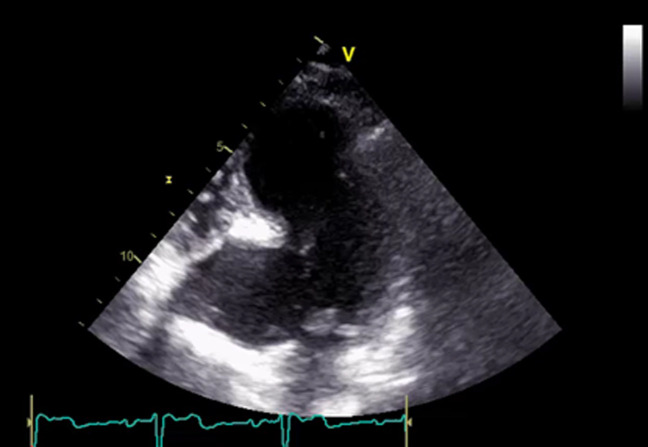
echocardiography in the apical 4 cavities view showing a giant calcified aneurysm of the basal inferoseptal wall

**Figure 4:**
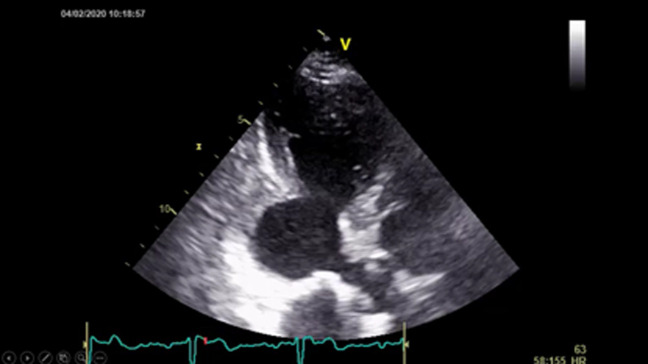
echocardiography in the apical 3 cavities view showing a giant calcified aneurysm extending to the basal inferoseptal wall

**Figure 5:**
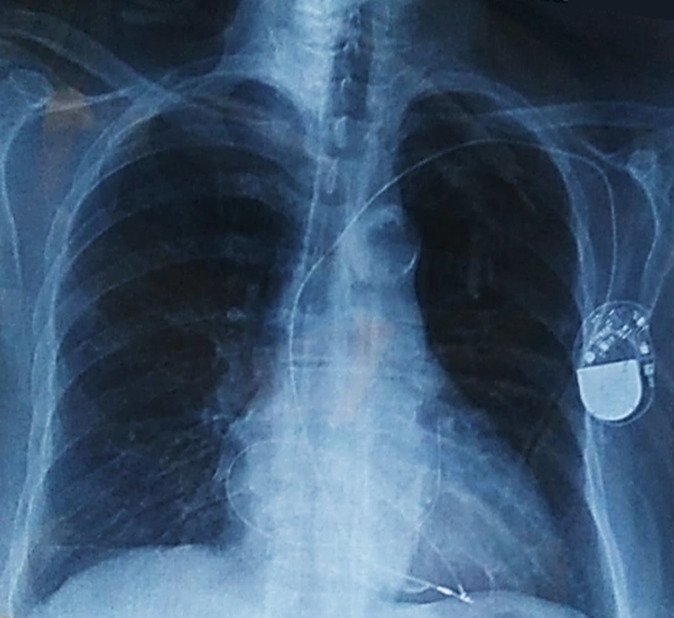
chest X-rays showing no cardiomegaly, and a second probe, cut and rolled up in the right ventricle

**Figure 6:**
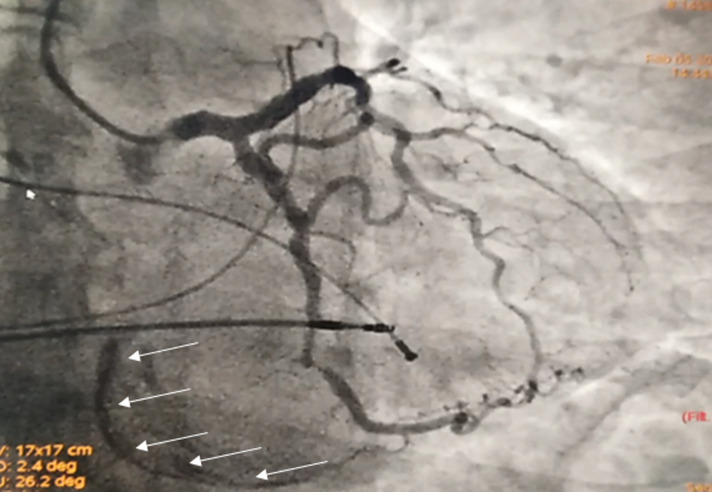
coronary angiography in the right oblique view. White arrows outline the calcified aneurysm

**Figure 7:**
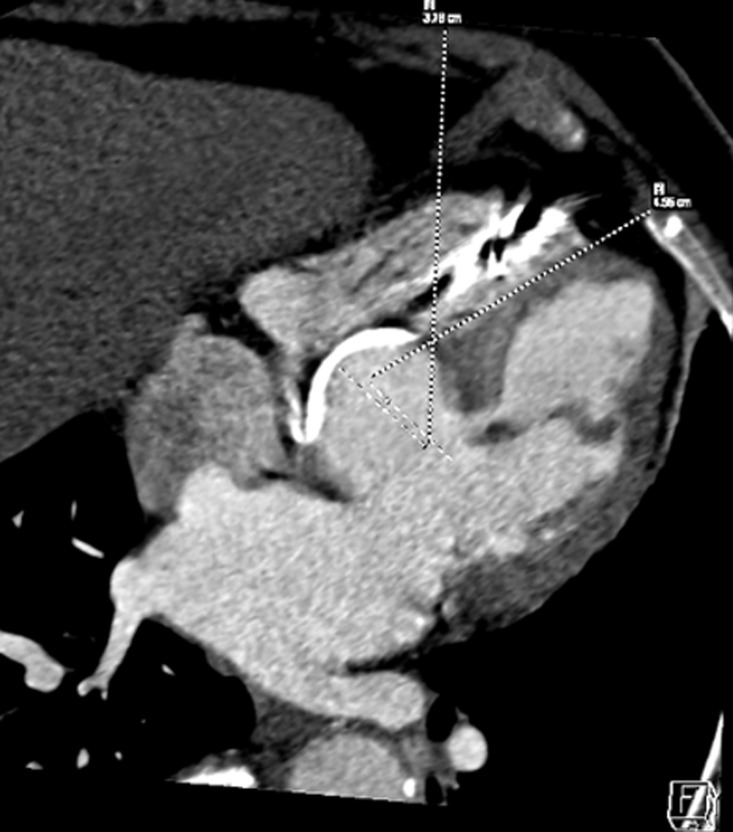
cardiac CT scan showing a heavily calcified aneurysm

An 82-year-old female patient was hospitalized for a life threatening arrhythmia. Medical history revealed hypertension and a complete atrioventricular (AV) block treated by a single-chamber pacemaker in 1996. The lead was changed few years later for an unknown reason. Ventricular tachycardia was the most probable diagnosis using the Brugada and Vereckei algorithms (Figure 1). Clinical exam, found a patient with cardiogenic shock. Urgent electrical cardioversion was successfully made, and the patient was put on IV amiodarone. The second electrocardiogram (ECG) showed Q waves in lead DIII with inverted T waves in leads DIII and arteriovenous fistula (AVF) and a first degree AV block (Figure 2). The patient described having palpitation and atypical chest pain few hours before her admission. Troponins were slightly elevated (0.06ug/l for a limit of 0.05ug/l) without any other biological abnormalities. Echocardiography showed non dilated left chambers, a preserved LV ejection fraction (visually estimated at 50-55%), a giant calcified basal inferior left ventricular aneurysm with a thrombus at its bottom and a non-severe mitral regurgitation (Figure 3, Figure 4). Chest X-rays showed no cardiomegaly, calcification at the bottom of the aneurysm and a second pacemaker lead, cut and rolled up in the right ventricle (Figure 5). The patient was transferred to the cath lab. Coronary angiography revealed: a right coronary artery with multiple lesions, occluded at its middle segment and a distal occlusion of the left circumflex artery. The calcifications of the aneurysm were also visible (Figure 6). Cardiac computerized tomography (CT) scan showed a large heavily calcified aneurysm measuring 45x38mm (Figure 7). The patient was put on anticoagulation, aspirin 100 mg/day, atorvastatin 40 mg/day, bisoprolol 5mg daily and candesartan 16mg daily. The patient had no impairment of her functional status, no ventricular arrhythmia, and no thromboembolic arterial events. The discussion was about whether to extract the second lead and to treat the aneurysm surgically especially with a Euroscore II of 7% but the patient refused any risky intervention. The patient was dischargedandno events were noted at a follow up of 4 months.

## Discussion

The exact incidence of LVA following MI is unknown and hard to precise. It was previously estimated that LVA develop in up to 35% of patients with Q wave MI. However, its incidence is clearly decreasing, and it currently occurs in approximately only 10% of patients [3]. This is because of major improvements in the management of MI. Approximately 80% of LVA are located in the anterior and/or apical walls, most commonly associated with left anterior descending artery occlusion [3] but any region may be concerned. In 10 to 15% of cases, LVA involve the inferior wall, basal inferior wall aneurysms constitute nearly 3% of all LVA while lateral aneurysms appear to be remarkably rare [4]. Calcified ventricular aneurysms are seldom observed in modern clinical practice [5] and a calcified basal inferior LVA is an even rarer coincidence.

To our knowledge this is the second case published, the only one found onafter a review of the literature with the words “calcified”, “basal”, “inferior”, “left ventricular aneurysm” and “LVA”, was published in 2018 [6]. Although aneurysms may be discovered accidentally on a routine echocardiographic exam, symptoms may include, angina or dyspnea due to systolic and diastolic dysfunction, ventricular arrhythmias leading to syncope, palpitation and sudden death and heart failure [3]. Thromboembolic events (stroke, peripheral vascular disease or MI) are unusual [3]. Transthoracic echocardiography is the gold standard because it gives an idea on the systolic and diastolic functions, the aneurysm size, the associated valvular lesions and the existence of a thrombus [7]. Once an aneurysm is suspected on echocardiography, the greatest diagnostic dilemma is differentiating between a true LVA and pseudoaneurysms (PA). A PA or false aneurysm is defined as a rupture of the left ventricular wall enclosed by a surrounding pericardium [2].

Other imaging modalities including computed tomographic angiography and magnetic resonance have been described, but are time-consuming, expensive, and not universally available [6]. Some factors are believed to be associated with a less survival rate in patients with LVA and these include age, class of heart failure, extent of coronary artery disease, mitral regurgitation, ventricular arrhythmia, aneurysm size, residual Left ventricular ejection fraction, left ventricular end diastolic pressure, and early development of LVA [8]. There is still no clear guidelines on how to treat left ventricular aneurysms. Surgery is preferred in the presence of recurrent angina, large aneurysm, congestive heart failure, rupture, recurrent embolism and recurrent arrhythmia [8] and for refractory cases (a class II a recommendation according to the American College of Cardiology/American Heart Association guidelines). The Surgical Treatment for Intracerebral Hemorrhage (STICH) trial concluded, however, that surgery is with no impact on functional class improvement, reduction in mortality, or hospitalization rates from cardiovascular disease [9]. The main goals of aneurysmectomy are generally to reduce the end diastolic volume, to maintain the ideal shape of the ventricle, and to avoid the occurrence of thrombogenesis [10] but the indications for calcified ventricular aneurysmectomy remain controversial. Medical treatment focuses on after load reduction, anti-anginal therapy, and anticoagulation in cases demonstrating substantial left ventricular dysfunction or thrombus formation.

## Conclusion

In conclusion, LVA are a common consequence of MI. Cases of calcified basal inferior LVA are very rare. Echocardiography is the best diagnostic exam and in the absence of clear guidelines, a case by case management is necessary.
